# Simple modification of trauma mechanism alarm criteria published for the TraumaNetwork DGU^®^ may significantly improve overtriage – a cross sectional study

**DOI:** 10.1186/s13049-018-0498-x

**Published:** 2018-04-24

**Authors:** Philipp Braken, Felix Amsler, Thomas Gross

**Affiliations:** 1 Kantonsspital Aarau Traumatology, Tellstrasse 25, CH-5001 Aarau, Switzerland; 2 Amsler Consulting, Gundeldingerrain 111, CH-4059 Basel, Switzerland

**Keywords:** Trauma team activation, Alarm criteria, Glasgow Coma Scale, Mechanism of injury, Overtriage, Undertriage, Injury Severity Score, TraumaNetwork DGU^®^, Emergency room

## Abstract

**Background:**

No consensus exists in the literature on the use of uniform emergency room trauma team activation criteria (ERTTAC). Today excessive over- or undertriage rates continue to be a challenge for most trauma centres. Application of ERTTAC, published for use in the German TraumaNetwork DGU^®^, at a Swiss trauma centre resulted in a high overtriage rate. The aim of the investigation was to analyse the ERTTAC in detail with the intention of possible improvement.

**Methods:**

The investigation included consecutive adult (age > 15 years) trauma patients treated at the emergency department of a level II trauma centre from 01.01.2013–31.12.2015. All data were collected prospectively. To identify over- and undertriage, patients with an Injury Severity Score (ISS) > 15 were defined as requiring specific emergency room (ER) management. ANOVA, Student’s t-test and chi-square analysis were used for statistical analysis with mean values ± standard deviation.

**Results:**

1378 adult injured (64% male) received ER trauma team treatment (mean age 48.3 ± 21.2 years; ISS 9.7 ± 9.6) during the observation period. Of those, 326 ER patients (23.7%) were diagnosed with an ISS > 15, which proved to be an overtriage of 76.3%. 80/406 trauma patients with an ISS > 15 were not referred to the ER, resulting in an actual undertriage rate of 19.7%, mainly because the criteria list was not observed. Effectively applying ERTTAC according to the protocol in all cases would have reduced undertriage to 2.0% (8/406). The most frequent trigger for trauma team activation was injury mechanism (65%). A simulation revealed that omitting the criterion ‘passenger of car or truck’ (*n* = 326) would have prevented overtriage in 257 cases, as such lowering overtriage rate to 62.4% and at the same time increasing undertriage by only 8 cases to 7.1%.

**Conclusion:**

Application of ERTTAC as published for TraumaNetwork DGU^®^ resulted in a lower undertriage but higher overtriage rate than recommended by the American College of Surgeons. Omitting the criterion ‘passenger of car or truck’ markedly improved overtriage with only a minimal increase in undertriage.

**Trial registration:**

NCT02165137; retrospectively registered 11. June 2014.

## Background

In the context of a quality improvement project on the treatment of major trauma in a level II trauma centre one of the objectives was to adequately standardize emergency room (ER) treatment. No generally accepted international standard or national recommendations for ER trauma team activation (ERTTA) criteria (ERTTAC) were found in the literature, on the contrary, published trauma triage protocols are highly divergent [1–6]. We chose the most up-to-date ERTTAC published by and recommended for use in the German TraumaNetwork DGU^®^ (Table 1) [7]. These criteria were developed from the evidence-based recommendations for the treatment of the severely injured as published by the German Society for Trauma Society (DGU®), but are less strict. For example, for the TraumaNetwork DGU^®^ ERTTAC a Glasgow Coma Scale (GCS) < 14 was chosen as an alert criterion for ERTTA instead of a GCS < 9 as given in the evidence-based recommendations. This principal decision for a more aggressive inclusion of trauma patients in the ER aimed to reduce the risk of missing severe trauma cases.

**Table 1:** ERTTAC* used for adult patients with suspected severe trauma

1) admission from external hospital		
2) injury mechanism	3) anatomic criteria	4) physiological criteria
proximal gunshot/stab wound	open thoracic injury	intubation
pedestrian vs. vehicle	unstable thorax injury	respiratory deficiency
passenger in vehicle: velocity ≥ 50 km/h or car body deformation ≥ 50 cm	severe abdominal injury	respiratory frequency < 10 or > 30/min
ejection from a vehicle	unstable pelvis fracture	SaO2 < 90%
death of a vehicle passenger	proximal amputation	systolic blood pressure < 90 mmHg
collision with rail vehicle	proximal vessel injury	GCS < 14
fall from ≥ 3 m	> 1 long bone fracture	
explosion	moderate/severe head injury	
entombment		

Emergency room trauma team activation criteria, ERTTAC; Glasgow Coma Scale, GCS; blood pressure RR; oxygen saturation SaO2; *as published for the TraumaNetwork DGU^®^ [7]

Following the introduction of ERTTAC at our institution, it was realized that the number of ER trauma cases was sizeable, indicating that too many minor trauma patients were being referred to the ER. Since we could not find any reports on over- and undertriage following the use of the ERTTAC published for the TraumaNetwork DGU^®^, we decided to investigate our experience in more detail.

This investigation had two major objectives:

First, to quantify over- and undertriage rates following the standard use of ERTTAC published for the TraumaNetwork DGU^®^ in a trauma centre and second, to identify single criteria that might be modified to decrease overtriage without the consequence of high undertriage.

## Methods

The teaching hospital is one of 12 dedicated trauma centres in Switzerland with a catchment area of about 2000 km^2^ with about 750,000 inhabitants. For reasons of quality control the level II trauma centre enters its data in the TraumaRegister DGU^®^, but does not participate in the TraumaNetwork DGU^®^ (www.traumanetzwerk-dgu.de), the latter comprising about 650 European hospitals.

### Study design

At the end of 2011, the ERTTAC published for the TraumaNetwork DGU^®^ were introduced for emergency room trauma team activation (ERTTA) at our hospital as part of a quality assurance programme [7]. The ERTTAC list (Table 1) was used in a standardized manner for all emergency trauma cases, i.e. if at least one of these criteria was fulfilled the dedicated interdisciplinary trauma team on call was activated by an appel circulaire to await the patient’s arrival in the emergency room. In contrast, trauma patients which did not meet the ERTAAC were primarily treated by single emergency nurses and specialists in the emergency department.

### Study setting

Trauma cases passing through the emergency department were controlled for injury severity at the end of hospitalization. Data management was executed by specifically trained study nurses who were not involved in the treatment of single cases. Included in this investigation (permission by the Cantonal Ethical Commission Aargau 27.3.2012) were all adult (defined as > 15 years of age) trauma emergency department cases from 1.1.2013–31.12.2015 who sustained an injury within 24 h prior to hospital admission (according to the criteria of the TraumaRegister DGU^®^) and who were either referred immediately to the ER after trauma team activation (independent from evaluated trauma severity) or were found to have an ISS > 15 at the end of hospitalization. All trauma ER cases were prospectively registered. Prehospital variables were extracted from the ambulance or helicopter documentation. Patients’ demographic data and other variables essential to determining all scores were collected prospectively on admission, whereby the first available value, either preclinical or on arrival at the hospital, was used for analysis.

### ERTTAC

The ERTTAC are subdivided into four groups, which are tailored to type of admission, injury mechanism, anatomic and physiological measures of adult patients (Table 1). If at least one ERTTA criterion is fulfilled, the patient should be treated in the ER. Because admission from an external hospital is a criterion per se, primarily admitted patients as well as secondary referrals from other hospitals to the ER were included in the study.

With regard to injury mechanism criteria, prospective registration included whether a criterion was met (yes or no) and, if yes, a description of which one. For detailed simulation analysis with modified criteria, subgroups of injury mechanism, e.g., car /truck passengers only or injury mechanism for non-car /truck passengers were specified accordingly. The detailed severe anatomic ERTTAC, i.e. specific clinical conditions such as open thorax etc., could not be evaluated in a prospective consecutive manner. As the emergency medical service (EMS) on scene for every patient in a standardised manner graded each observed injury lesion to one of in total nine anatomic regions into low, moderate or severe, for this investigation and based on the ERTTAC list (Table 1), the anatomic ERTTA criterion was defined as fulfilled if injuries to at least two of these regions were graded as moderate by the EMS service or at least one as severe (EMS injury).

### Scoring

The calculation of scores was undertaken according to the literature: Abbreviated Injury Severity (AIS) [8]; Injury Severity Score, ISS [9]; Glasgow Coma Scale, GCS [10]; Revised Injury Severity Score, RISC [11].The calculation of injury severity scores was undertaken with the maximum information available on hospital discharge.

An Injury Severity Score (ISS) > 15 was used to define ‘severe trauma’ as proposed by the American College of Surgeons (ACS) [12]. This definition aimed to identify the severely injured requiring ER trauma team treatment at a trauma centre more specifically. Findings from a previous study showed that this approach leads to a reduction in mortality [13].

### Over- and undertriage and attribution rates

In accordance with the American College of Surgeons [12], overtriage in the context of this paper was defined as the number / percentage of patients received *ER trauma team treatment* within 24 h after injury without presenting ‘severe trauma’, i.e. an ISS > 15 (as coded based on the maximum information available at the end of hospitalization). Undertriage was defined as the number / percentage of patients who presented at the emergency department without ER trauma team treatment, even though they were found to have an ISS > 15 at the end of hospitalization. With regard to the indication for ERTTA the recorded percentages for false and correct positive classification (i.e., activation of ER trauma team) as well as false and correct negative classification (i.e., *non-*activation of ER trauma team) of cases are given in relation to the ERTTAC combinations. Data were given for both, *actual* ER trauma team treatment and *theoretical* ERTTA rates.

### Statistics

For the calculation of over- and undertriage the statistical formula as defined by Peng et al. was used [14]. With regard to the calculation of the over- and undertriage-rates missing information was treated as having no direct bearing on trauma activation (under the assumption that those values would have been measured in critical cases).

The results are presented as means ± standard deviation (SD) if not stated otherwise. All statistical tests were two-tailed. Student’s t-test was used for comparison of means in normally distributed data of continuous variables; ANOVA for similar criteria in 3 or more unpaired subsamples. Chi-square analysis was used to test categorical data. Data were analyzed using SPSS™ for Windows 24 (Armonk, NY: IBM Corp, USA), and a *p* value < 0.05 was considered significant.

## Results

### Over- and Undertriage

A total of 1378 adult trauma patients underwent ER trauma team treatment during the study period (Table 2). 326 of these patients (23.7%) had an ISS > 15; i.e., the actual overtriage rate was 76.3%. During the same time period *n* = 80 trauma patients with an ISS > 15 arrived at the emergency department without ERTTA, i.e., the actual undertriage rate was 19.7% (80 out of 406). 72 of these 80 patients in effect fulfilled at least one ERTTA criterion, i.e. the undertriage for this group would have been 2.0% if the ER trauma team had been activated according to the protocol. These eight patients presented with an age range of 17 to 79 years, an ISS from 17 to 29 and a RISC (Revised Injury Severity Score) from 1.4 to 62.5. None of them died. Considering all trauma patients that underwent ER trauma team treatment, 913 of the 1052 patients with ISS ≤ 15 fulfilled at least one ERTTA criterion, i.e., the *theoretical* overtriage was found to be 87%. On the other hand, 15 of the 326 patients with an ISS > 15 did not fulfil any criteria for ERTTA, i.e., the *theoretical* undertriage rate was found to be 4.6%.

**Table 2:** Characteristics of patients

	cases in the ER				cases not in the ER	
		ISS ≤ 15	ISS > 15		ISS > 15	
	(*n* = 1378)	(*n* = 1052)	(*n* = 326)		(*n* = 80)	
	Mean ± SD	Mean ± SD	Mean ± SD	*p*	Mean ± SD	*p*
	N (%)	N (%)	N (%)		N (%)	
male	879 (63.8%)	658 (62.5%)	221 (67.8%)	0.09	48 (60%)	0.20
female	499 (36.2%)	394 (37.5%)	105 (32.2%)		32 (40%)	
age at accident (years)	48.3 ± 21.2	45.7 ± 20.4	56.6 ± 21.6	< 0.001	68.7 ± 19.1	< 0.001
ISS	9.7 ± 9.6	5.3 ± 4.3	23.9 ± 7.9	< 0.001	20.3 ± 4.5	< 0.001
NISS	12.5 ± 12.3	7.1 ± 6.5	29.7 ± 11	< 0.001	25.4 ± 7.2	0.001
AIS1	1.3 ± 1.5	0.9 ± 1	2.7 ± 1.8	< 0.001	3.7 ± 1.3	< 0.001
AIS2	0.2 ± 0.6	0.1 ± 0.5	0.5 ± 0.9	< 0.001	0.5 ± 0.9	0.99
AIS3	0.8 ± 1.3	0.4 ± 0.9	1.9 ± 1.6	< 0.001	0.6 ± 1.2	< 0.001
AIS4	0.4 ± 0.9	0.2 ± 0.6	0.9 ± 1.3	< 0.001	0.2 ± 0.6	< 0.001
AIS5	0.8 ± 1.2	0.5 ± 1	1.4 ± 1.4	< 0.001	0.7 ± 1.1	< 0.001
AIS6	0.6 ± 0.6	0.6 ± 0.5	0.4 ± 0.7	< 0.001	0.3 ± 0.4	0.03
1st GCS	13.3 ± 3.4	14 ± 2.5	11.1 ± 4.7	< 0.001	13.8 ± 2.1	< 0.001
lowest SaO2 (%)	94.1 ± 10.1	94.5 ± 10.2	92.9 ± 9.6	0.06	93.3 ± 5.3	0.85
lowest systolic BP (mmHg)	133 ± 28	134.6 ± 27	128.2 ± 30.5	0.004	136 ± 21.7	0.20
RISC (%)	7.7 ± 16.8	3.1 ± 5.9	22.4 ± 28.3	< 0.001	22.8 ± 20	0.90
hospital mortality	96 (7%)	17 (1.6%)	79 (24.2%)	< 0.001	7 (8.8%)	0.002

emergency room, ER; (new) injury severity score, (N)ISS; abbreviated injury scale, AIS (AIS 1: head & neck; etc.); blood pressure, BP; blood oxygen saturation, SaO2; Revised Injury Severity Score, RISC; Glasgow Coma Scale, GCS

### Simulation models

Several simulation models were tested to further improve these observed rates. Table 3 gives the resulting theoretical over- and undertriage data with regard to an ISS > 15 for different single and combined ERTTAC.

**Table 3:** Simulation of theoretical over- and undertriage according to different single and combined ER trauma team activation criteria

ERTTAC		ISS ≤ 15	ISS > 15	total	*P*	over-triage	under-triage
trauma patients treated in the ER	N	1052	326	1378			
	%	76.3%	23.7%	100.0%			
Transfer criterion							
transfer from external hospital	no	936	253	1189	< 0.001	116/1052	253/326
	yes	116	73	189		11.0%	77.6%
	%	11.0%	22.4%	13.7%			
Mechanism of injury							
mechanism of injury (traffic)	no	513	199	712	< 0.001	539/1052	199/326
	yes	539	127	666		51.2%	61.0%
	*%*	51.2%	39.0%	48.3%			
mechanism of injury (car /truck passenger only)	no	726	288	1014	< 0.001	326/1052	288/326
	yes	326	38	364		31.0%	88.3%
	%	31.0%	11.7%	26.4%			
mechanism of injury (traffic, except car /truck passenger)	no	839	237	1076	0.008	213/1052	237/326
	yes	213	89	302		20.2%	72.7%
	%	20.2%	27.3%	21.9%			
fall from height ≥ 3 m	no	921	267	1188	0.01	131/1052	267/326
	yes	131	59	190		12.5%	81.9%
	%	12.5%	18.1%	13.8%			
penetrating injury (chest, abdomen)	no	1020	314	1334	0.56	32/1052	314/326
	yes	32	12	44		3.0%	96.3%
	%	3.0%	3.7%	3.2%			
Combined mechanism of injury							
mechanism of injury (penetrating, traffic, fall ≥ 3 m)	no	352	130	482	0.034	700/1052	130/326
	yes	700	196	896		66.5%	39.9%
	%	66.5%	60.1%	65.0%			
mechanism of injury without car criteria (penetrating, traffic, fall ≥ 3 m)	no	677	168	845	< 0.001	375/1052	168/326
	yes	375	158	533		35.6%	51.5%
	%	35.6%	48.5%	38.7%			
Anatomic criterion							
EMS injury	no	882	194	1076	< 0.001	170/1052	194/326
	yes	170	132	302		16.2%	59.5%
	%	16.2%	40.5%	21.9%			
Physiological criteria							
Intubation before arrival in ER	no	1003	235	1238	< 0.001	49/1052	235/326
	yes	49	91	140		4.7%	72.1%
	%	4.7%	27.9%	10.2%			
intubation in ER	no	1025	285	1310	< 0.001	27/1052	285/326
	yes	27	41	68		2.6%	87.4%
	%	2.6%	12.6%	4.9%			
SaO2 < 90%	no	981	282	1263	< 0.001	71/1052	282/326
	yes	71	44	115		6.7%	86.5%
	%	6.7%	13.5%	8.3%			
systolic (BP < 90 mmHg)	no	1028	310	1338	0.02	24/1052	310/326
	yes	24	16	40		2.3%	95.1%
	%	2.3%	4.9%	2.9%			
1st GCS < 14	no	897	178	1075	< 0.001	155/1052	178/326
	yes	155	148	303		14.7%	54.6%
	%	14.7%	45.4%	22.0%			
1st GCS < 9	no	992	236	1228	< 0.001	60/1052	236/326
	yes	60	90	150		5.7%	72.4%
	%	5.7%	27.6%	10.9%			
Combined physiological criteria							
physiological criteria (1st GCS < 14, intubation, SaO2 < 90%, BP < 90 mmHg)	no	821	136	957	< 0.001	231/1052	136/326
	yes	231	190	421		22.0%	41.7%
	%	22.0%	58.3%	30.6%			
physiological criteria (1st GCS < 9, intubation, SaO2 < 90%, BP < 90 mmHg)	no	888	164	1052	< 0.001	164/1052	164/326
	yes	164	162	326		15.6%	50.3%
	%	15.6%	49.7%	23.7%			
Overall combined criteria							
all criteria: 1st GCS < 14	no	139	15	154	< 0.001	913/1052	15/326
	yes	913	311	1224		86.8%	4.6%
	%	86.8%	95.4%	88.8%			
all criteria: 1st GCS < 9	no	167	24	191	< 0.001	885/1052	24/326
	yes	885	302	1187		84.1%	7.4%
	%	84.1%	92.6%	86.1%			
all criteria: 1st GCS < 14; without car criteria	no	396	23	419	< 0.001	656/1052	23/326
	yes	656	303	959		62.4%	7.1%
	%	62.4%	92.9%	69.6%			
all criteria: 1st GCS < 9; without car criteria	no	430	35	465	< 0.001	622/1052	35/326
	yes	622	291	913		59.1%	10.7%
	%	59.1%	89.3%	66.3%			

*ER* emergency room, *ERTTAC* emergency room trauma team activation criteria, *ISS* injury severity score, *BP* blood pressure, *SaO2* blood oxygen saturation, *GCS* Glasgow Coma Scale, *EMS injury* emergency medical service’s grading of injury severity: at least one grade severe or two moderate injuries; all criteria, mechanism, transfer, EMS injury, physiological

### Injury mechanism

**Fig. 1:**
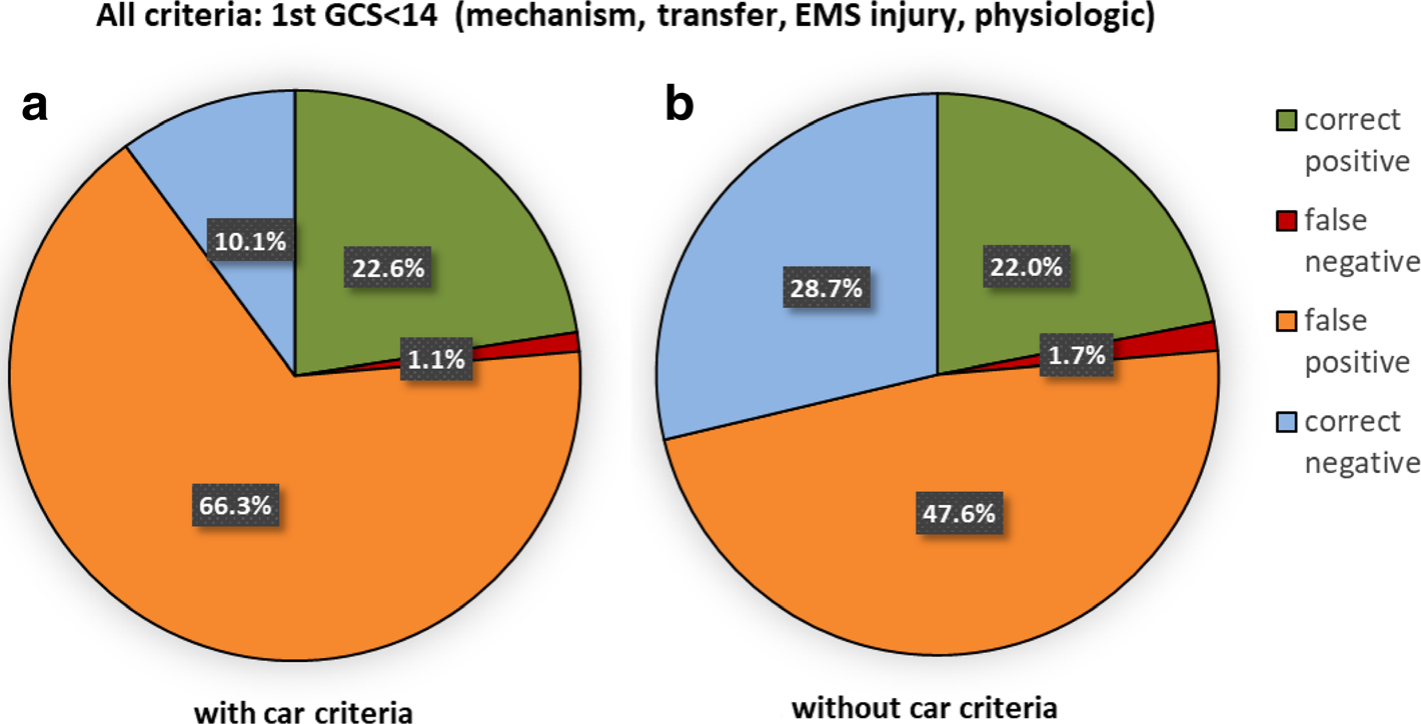
Sensitivity and specificity of ERTTA for the mechanism criterion ‘car/truck passenger’. Emergency room trauma team activation (ERTTA). Percentage of false and correct positive as well as false and correct negative classification of cases with regard to ERTTA with versus without the mechanism criterion ‘car/ truck passenger’

The most frequent cause for ERTTA was the injury mechanism: 65% of all patients fulfilled at least one mechanism criterion, 48% of whom had been in traffic accidents. Of the 1052 patients with an ISS ≤ 15, 539 had been in a traffic accident (51%) whereas this was only the case for 127 of the 326 patients with an ISS > 15 (39%; *p* < 0.001). If “traffic” were the only criterion for patients to be treated in the ER, the resulting overtriage would have been 51.2% and the undertriage 61.0%. 26% of traffic injured (*n* = 364) were car or truck passengers, more frequently in the ISS ≤ 15 group (*n* = 326, 31%) than in the ISS > 15 group (*n* = 38, 12%). For the overall combined criteria (with first GCS < 14) as it was used in our hospital, the overtriage was 86.8% and the undertriage 4.6%. If the mechanism criterion “car/ truck passenger” were not on the ERTTAC list, overall overtriage would have decreased by 257 cases (24.4%) down to 62.4%, whereas undertriage would have increased by 8 cases (2.5%) to 7.1%. The percentages for false and correct positive as well as false and correct negative classification of patients are given in Fig. 1.

### Physiological criteria

The criterion first GCS < 14 was observed in 22% of cases (*n* = 303). Leaving all other criteria unchanged but adjusting the first GCS criterion to < 9 would have reduced overtriage by 28 cases (− 2.2%, from 86.6% to 84.1%) with undertriage simultaneously increasing by 9 cases (+ 2.5%; from 4.6% to 7.4%).

## Discussion

To our knowledge this 3 year pilot study at a Swiss trauma centre is the first evaluation of the daily use of the ERTTAC as published for the TraumaNetwork DGU^®^ [7].

We obtained four major results: (1) Based on a definition of ISS > 15, rates for overtriage were 76% and for undertriage 20%, respectively. (2) If ERTAC would have been administered correctly, the undertriage would have been 4.6%. (3) If the critical GCS-criterion would be changed from < 14 to < 9, overtriage would decrease by 1.7% and undertriage increase by 1.1%. (4) If the trauma mechanism criterion for all car and truck passengers would be omitted, the overtriage rate would decrease by 24% (*n* = 257), while the undertriage rate would increase by 2.5% (*n* = 8).

1. With regard to *actual over- and undertriage, v*erifying the use of these ERTTAC based on a definition of ISS > 15 for severe trauma resulted in 76% overtriage and 20% undertriage at our institution. Both rates are higher than recommended by the American College of Surgeons (ACS). In 2014 the ACS revised its 2006 recommendations from 30 to 50% for overtriage and < 5–10% for undertriage to < 25–35% and < 5%, respectively [12]. The over- and undertriage rates found in this study are well within the range of data published from other level I and II trauma centres in the US and Europe, with reported overtriage rates varying between 23% [15] and 91% [16], and undertriage rates between 1% [17] and 32% [18].
  For this evaluation, we utilized the most commonly used definition of the ACS for the severely injured with an ISS > 15 to identify who should be treated at a trauma centre [19–21]. Some US-American studies recommend criteria additional to an ISS > 15, e.g. the urgent need for intervention or ICU treatment, to determine trauma centre need [22, 23]. Apart from the exact definition of trauma centre need, there is an ongoing debate as to whether or not all the identified patients automatically require ER trauma team activation. Most studies do not report details on the latter [19, 20, 24], whereby the official 2017 Swiss Trauma Board recommendations combine the indication for trauma centre with subsequent ER trauma team treatment.
2. With regard to *theoretical over- and undertriage,* only 15 of 326 patients with an ISS > 15 did not meet the TraumaNetwork DGU^®^ ERTTAC used in this investigation, yielding a *theoretical undertriage* rate of 4.6% if the criteria were applied properly in all cases. Such a rate would correspond well with the recent recommendations of the ACS [12]. Of the 80 cases not treated in the ER, even though their ISS was > 15, only 8 (10%) did not fulfil at least one ERTTA criterion. The theoretical undertriage for this group would have been 2.0%. In contrast, the *theoretical overtriage* rate of 87% at our institution would have been higher than found in reality. A high overtriage rate in daily practice often results from the criterion “gut feeling” [21] or a less accurate evaluation by paramedics compared to anaesthesiologists [25]. A study from France [24] demonstrated improved overtriage by strictly following triage protocols. Fitzharris et al. [26] demonstrated a 74% adherence rate to hospital protocols for triage for about 58,000 urban patients transported to trauma centres in New South Wales, Australia, and an even better adherence (85%) for cases where all ERTTAC were fulfilled. Overall, it seems difficult to uncover the reasons for single cases of non-adherence, which appear numerous and may be related to preclinical statements and/or to first clinical registration. Fitzharris et al. showed that although the triage protocol offered clear instructions, paramedics interpreted injury severity and treatment requirements differently [26]. Additionally, adherence was reported lower for female patients, older patients and less trained paramedics. In paediatric patients Escobar et al. [27] lowered the undertriage rate from 15% to 5% by applying a standardized procedure during patient registration to check whether ERTTAC were fulfilled. Empowering nurses to initiate trauma team activation was a crucial step to diminish undertriage. At our hospital an ED nurse on duty was responsible for ERTTA according to the protocol. Given our preliminary findings, the first step towards achieving the theoretically possible, low undertriage rate is to further increase adherence to the ERTTAC list. If other centres confirm our pilot results and manage to strictly implement these ERTTAC as published for the TraumaNetwork DGU^®^ in daily routine, they may expect sufficiently low undertriage rates in relation to ACS recommendations.
3. *With regard to the adjustment of the GCS-criterion,* to improve overtriage rates we were interested in how far simple adaptations of ERTTAC might lower the overtriage rate without relevantly compromising undertriage. First of all, we hypothesized a major effect due to modifying the GCS from < 14 to < 9, given the better evidence for the latter in the literature as a predictor of severe trauma [3, 28]. Nationwide studies from the US demonstrated that about 40% of undertriage cases for major trauma detection (ISS > 15) were TBIs [29]. Unexpectedly, the specially designed simulation tests on our data revealed only a slight improvement in overtriage expressed as a decrease of 2.2% (*n* = 28). Concurrently, undertriage increased by 2.5% (*n* = 8). Newgard et al. found GCS to be the best predictor for an ISS > 16, independent of patient age [30, 31]. With regard to the ACS triage criteria the required triage protocol sensitivity of 95% was not met by comparative testing of varying GCS ERTTAC. In contrast, Norwood et al. [28] strengthened the GCS ERTTAC from< 15 to < 13 and ultimately reported a deterioration of results for both over- and undertriage. The attempt to improve such over- and undertriage rates combined with simultaneous simplification of GCS application by using only its motor component [32] resulted in a mere 1.7% decrease in overtriage with a concurrent increase in undertriage of 1.1%. Given our results in the context of the current literature we saw no sufficient argument as to why we should change our ERTTAC with regard to the GCS.
4. *With regard to the adjustment of the trauma mechanism-criterion, s*imulation testing revealed a simple way to reduce the relatively high overtriage of about 80% in our setting by modifying only one parameter: Excluding the trauma mechanism criterion for all car and truck passengers was shown to decrease the overtriage rate by 24% (*n* = 257) without relevantly increasing undertriage (2.5%; *n* = 8). Several authors identified the problem of high overtriage as a result of relatively non-specific trauma mechanism criteria and reported overtriage rates over 90% if these criteria were used in isolation [16, 33]. In 2006 the ACS restricted their injury mechanism criteria, for example, by excluding the deformity criterion or adapting speed limits. Implementing these stricter criteria in a study including data from three level I trauma centres, Lerner et al. reported on a reduction of overtriage from 34% to 23% with a simultaneous increase of undertriage from 3.3% to 4% [15]. Uleberg et al. found that high energy trauma per se did not justify trauma team activation in asymptomatic patients [33]. Totally omitting injury criteria from the ERTTAC list was shown to result in a decrease of overtriage by 22–35% with a simultaneous increase of undertriage by 1–3% [17, 34]. Lerner et al. reported that ERTTAC such as death of another passenger in the same vehicle, height of fall and pre-hospital rescue time best predicted the need for trauma centre resources [16]. A fall from a height of over 5 m correlated with more severe trauma and resulted in higher undertriage if not used as an ERTTA criterion [20]. In our cohort this was already true for a fall from a height of over 3 m. Davidson et al. found vehicle criteria to correlate with an ISS > 15 mostly in elderly patients > 55 years of age [35]. Brown et al. concluded that even though physiological ERTTAC generally appear to be predictive of an ISS > 15 and anatomic criteria of the need for an operative intervention, the combination of both will result in an undertriage of up to 50% if not restricted by additional injury mechanism criteria [36]. So far there is no consensus in the literature on the use of mechanism criteria, especially regarding traffic injuries. Most authors conclude that if these are used alone, they have little predictive value in determining the need for trauma centre care [17, 34]. An adaptation of these ERTTAC to the progress of security techniques by the automotive industry appears overdue and given our finding that for almost every fourth trauma patient unnecessary ERTTA could be avoided by a simple restriction of injury mechanism criteria, we would opt for a corresponding adaptation of the ERTTAC published for the TraumaNetwork DGU^®^. Future improvement might be achieved by the use of advanced automatic crash notification [37] or more complicated specific computer algorithms [38] as indicated by recent studies.

### Limitations

The findings of this pilot monocenter study are limited to the cohort under investigation and the simulation of adapted ERTTAC. The results have to be evaluated by other groups and should additionally include a higher percentage of penetrating trauma or more severely injured patients. This constraint should not relevantly compromise our main findings, which are based on the combined analysis of ERTTAC, mostly with regard to traffic. As a next step towards further evaluation, we decided to keep separate records prospectively for each ERTTAC subgroup (external admission, injury mechanism, anatomic and or physiological criteria) if a criterion led to trauma team activation. Unfortunately, due to staff restrictions in most ER emergency situations, yet in the next future a preferable complete prospective registration of detailed specific anatomic criteria in every patient for further evaluation will not be possible in our hospital. Even though strict internal guidelines existed, and users were trained accordingly, we cannot guarantee that in all cases with an ISS < 15 ERTTAC were effectively used. This has also been reported by other authors with adherence rates to stipulated ERTTAC being as low as 74% despite clear instructions [26]. It is well known in this context that malcompliance in regard to protocol adherence, for example, by using the criterion “gut feeling” will probably always play a role in trauma team activation. Since not all severely injured patients necessarily met one of the ERTTAC, there might be some residual excuse for such behaviour as long as existing guideline criteria are unable to differentiate more reliably.

## Conclusions

In the literature no consensus exists as to which trauma patients should be treated by the emergency room trauma team. Applying the ERTTAC published for the TraumaNetwork DGU^®^, we observed higher under- and overtriage rates than recommended by the American College of Surgeons. Undertriage was mainly caused by non-compliance to the triage protocol. If the ERTTAC had been applied properly in all cases, the resulting theoretical undertriage rate would conform well to the recommendations of the American College of Surgeons. According to our data excluding the trauma mechanism criteria for car and truck injuries could improve the overtriage rate importantly without relevantly increasing undertriage of the severely injured. If other centres confirm these pilot results and manage to strictly execute the ERTTAC as published for the TraumaNetwork DGU^®^ in daily routine, sufficiently low undertriage rates in relation to the ACS recommendations may be expected.

## Abbreviations

ACS: American College of Surgeons
AIS: Abbreviated Injury Severity Scale
DGU^®^: Deutsche Gesellschaft für Unfallchirurgie (German Society for Trauma Surgery)
EMS: emergency medical service
ER: emergency room
ERTTA: emergency room trauma team activation
ERTTAC: emergency room trauma team activation criteria
GCS: Glasgow Coma Scale
ISS: Injury Severity Score
RISC: Revised Injury Severity Score
